# Conditional reduction of the loss value versus reinforcement learning for biassing a de-novo drug design generator

**DOI:** 10.1186/s13321-022-00643-2

**Published:** 2022-09-27

**Authors:** Mohamed-Amine Chadi, Hajar Mousannif, Ahmed Aamouche

**Affiliations:** 1 Laboratoire Ingénierie des Systems Informatiques (LISI), Department of Computer Science, Faculty of Sciences Semlalia, Cadi Ayyad University, 40000 Marrakech, Morocco; 2 Laboratoire Ingénierie des Systèmes et Applications (LISA), Ecole Nationale des Sciences Appliquées de Marrakech, Cadi Ayyad University, BP 575, Avenue Abdelkrim Khattabi, 40000 Marrakech, Morocco

**Keywords:** Deep molecular generation, Biassing techniques, Deep reinforcement learning, Conditional loss reduction

## Abstract

Deep learning has demonstrated promising results in de novo drug design. Often, the general pipeline consists of training a generative model (G) to learn the building rules of valid molecules, then using a biassing technique such as reinforcement learning (RL) to focus G on the desired chemical space. However, this sequential training of the same model for different tasks is known to be prone to a catastrophic forgetting (CF) phenomenon. This work presents a novel yet simple approach to bias G with significantly less CF than RL. The proposed method relies on backpropagating a reduced value of the cross-entropy loss used to train G according to the proportion of desired molecules that the biased-G can generate. We named our approach CRLV, short for conditional reduction of the loss value. We compared the two biased models (RL-biased-G and CRLV-biased-G) for four different objectives related to de novo drug design.

CRLV-biased-G outperformed RL-biased-G in all four objectives and manifested appreciably less CF. Besides, an intersection analysis between molecules generated by the RL-biased-G and the CRLV-biased-G revealed that they can be used jointly without losing diversity given the low percentage of overlap between the two to further increase the desirability. Finally, we show that the difficulty of an objective is proportional to (i) its frequency in the dataset used to train G and (ii) the associated structural variance (SV), which is a new parameter we introduced in this paper, calling for novel exploration techniques for such difficult objectives.

## Introduction

The cost and time of developing a drug and getting it to the market are estimated to be around 2.6$ billion and ten years, respectively [7]. In this respect, many researchers proposed deep learning as a potential solution to mitigate this problem, with many successful demonstrations presented [16]. In most contributions related to deep learning-based drug design, a similar pipeline is proposed: training a general model (G) to learn the building rules of valid molecules, then using a biassing technique to focus G on the desired chemical space.

One widely used approach for biassing the general model is transfer learning (TL) [8, 27, 29, 33, 34]. This consists of fine-tuning the pretrained G using new training data (molecules) that possess mainly the desired properties. Although it showed a consistently good performance, TL has limitations. The main drawback of TL is its reliance on data for fine-tuning, which may not be available, especially when many constraints must be satisfied. For instance, the number of molecules in the Chembl21 dataset [4]; often used for training deep learning-based drug design models, is approximately 1.6 million. The number of molecules with lipophilicity (logP) satisfying the first criteria of the modified Lipinski rule of five, the rule of three [3, 9], which states that orally bioavailable compounds are most likely to have a logP ≤ 3, is 315 k. However, the number of molecules that satisfy all five criteria of the Lipinski rule of three, that is, logP ≤ 3, molecular weight ≤ 480 g/mol, hydrogen bond acceptors ≤ 3, hydrogen bond donors ≤ 3, and rotational bonds ≤ 3, is only 72 k out of the 1.6 million. The more constraints are added, the more data for fine-tuning tend to be unavailable.

Another well-established method that is widely used for biassing G is reinforcement learning (RL) and deep RL [26]. The advantage of RL is that it is data-free, meaning that it does not depend on existing data. Indeed, the desired properties are encoded in a reward function that should be maximized by the general model, thus focusing on the desired chemical space. However, although RL has been adopted in many contributions in this regard [1, 5, 12, 18, 20, 23, 24], the reproducibility of the presented results is often difficult. This is caused by the many inherent sources of non-determinism and stochasticity in RL, the relatively high sensitivity to hyperparameters tuning, and the various hand-engineered rewards [17].

Moreover, this sequential/continual training of the same model for different tasks is known to be prone to a catastrophic forgetting (CF) phenomenon, where the model tends to forget the necessary knowledge learned for a previous task when training for a new one [10].

In this work, we introduce a novel yet simple method for biassing the general model G named CRLV, short for conditional reduction of the loss value. The primary goal of CRLV is to play the role of RL, i.e., a data-free biassing technique, but with significant mitigation of the RL limitations discussed, mainly the problem of catastrophic forgetting during the biassing process, and hopefully, to improve the performance in comparison with RL in terms of several metrics such as the number of molecules with desirable properties as well as the related novelty and diversity.

The rest of this paper is organized as follows: “Methods” section presents the inner-working intuition of the CRLV approach and the setting of the comparative experiments. “Results and discussion” section reports the results of the conducted experiments. Finally, “Conclusion” section discusses the results for further insights and principal conclusions.

## Methods

### Molecular generation: data and the general model

In a deep learning-based molecular generation, one of the most frequently used molecular representations is the simplified molecular line-entry system (SMILES), a one-line string representation composed of 72 characters. This includes letters for atoms (e.g., C for carbon) and symbols for the molecule's structure (e.g., = and # for double and triple bonds, respectively). In this regard, deep learning-based molecular generation can be considered a classification problem. The input is one character of the SMILES representing a molecule in a training dataset, and the output is a Softmax probability distribution (Eq. 1) overall characters, which we then sample from. The loss is then calculated as the cross-entropy (Eq. 2) between the predicted output character and the actual output (i.e., the next character in the input SMILES).

1 $$P(yp)=\frac{{e}^{(yp)}}{\sum_{j=1}^{k}{e}^{(yp)}}$$

2 $$CE(yp, yr)=-\frac{1}{k} \sum_{i=1}^{k}yr*log(yp)$$

where yp and yr are the predicted and actual output, respectively, and k is the number of classes (characters).

We herein use a similar pipeline, with an added temperature parameter (T) to the Softmax equation. Thus, Eq. 1 is transformed into Eq. 3 described below.

3 $$P(yp)=\frac{{e}^{(yp/T)}}{\sum_{j=1}^{k}{e}^{(yp/T)}}$$

The temperature parameter (T) is used to tune the output distribution of the generative model: higher values of T tend to equalize the probability of all characters, while lower values of T increase the probability for characters in the higher confidence interval and decrease the probability for other characters.

**Fig. 1:**
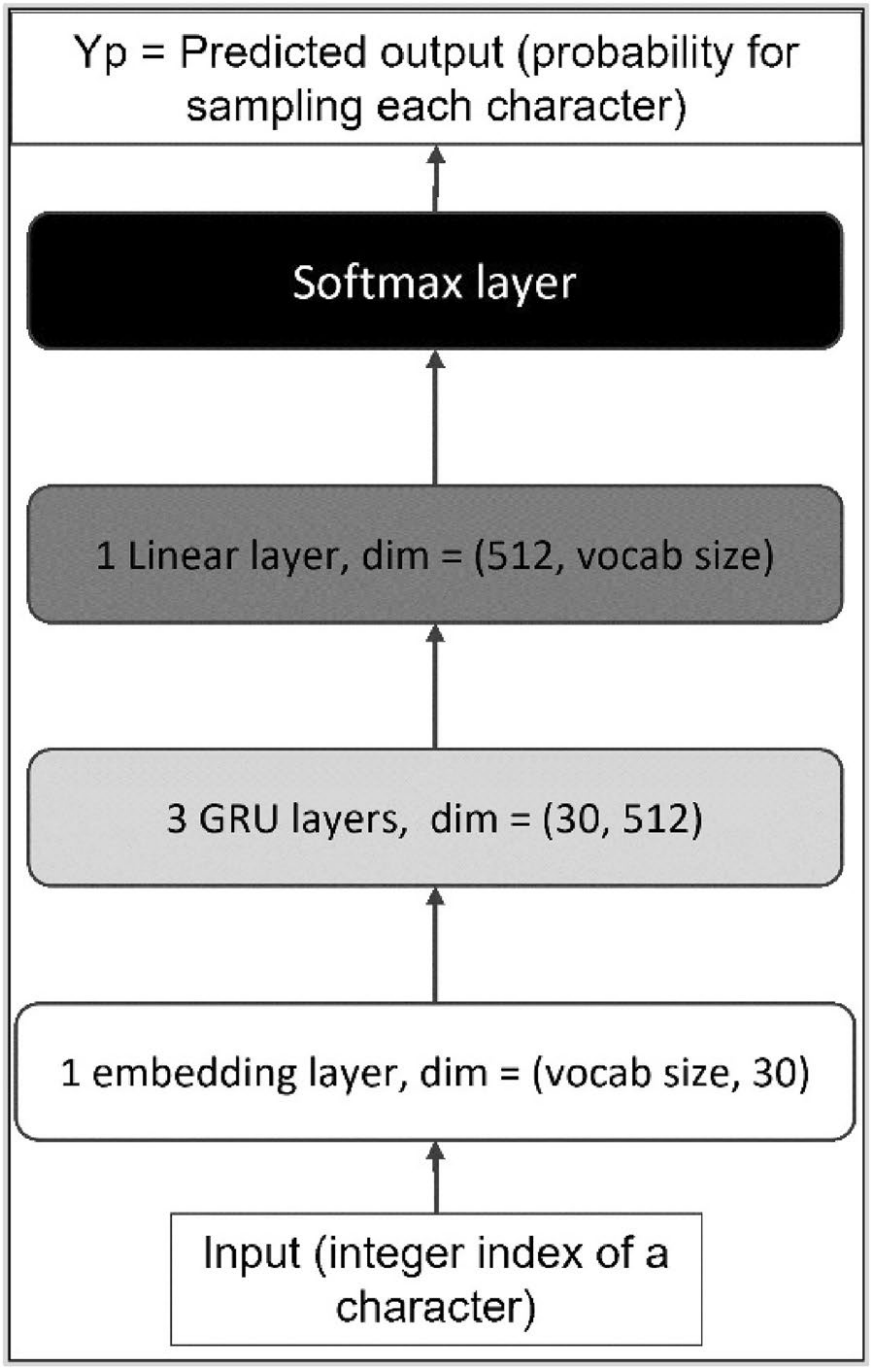
The architecture of the general model G

For the molecular generator (G), we used a recurrent neural neutral (RNN) model with gated recurrent units (GRU) [6]. The model’s architecture consists of (i) one embedding layer (dimension: vocabulary size, embedding size), (ii) three GRU layers (dimension: 512 units), (iii) and a linear layer (dimension: vocabulary size) as illustrated in Fig. 1.

The Chembl21 dataset [4] was used for training. We excluded molecules whose size (i.e., number of characters in the SMILES representation) is more than 100; given their negligible presence in the dataset (only ~ 2.7% of molecules have a size more than 100), we were left with 1.49 million molecules. All molecules were subject to a tokenization procedure: each character in a SMILES is represented by a unique integer (please see Appendix A and B for more details on data preparation and the pseudocode for training G). Finally, we launched the training loop for 2500 epochs, with a learning rate of 0.0005 using the Adam optimizer [11]. The learning curve of G is illustrated below in Appendix C: Fig. 7.

### Biassing G using RL

Reinforcement learning (RL) is an area of machine learning (ML) that mainly treats the optimization of decision-making problems. In RL, an agent (learning model) interacts with an environment by taking actions that should maximize a feedback reward. In the last decade, RL coupled with deep learning (i.e., deep RL) has demonstrated unprecedented success in decision-making problems, ranging from video and board games [14, 30] to healthcare and drug design [35, 36]. Deep RL has shown the ability to handle problems with a vast exploration space such as the game of Go, which was considered an impossible task because of the theoretical complexity of more than 10^140^ (Van den [32]. This makes it suitable for the present task (i.e., drug design), given the large chemical space of drugs estimated to be more than 10^60^ [21].

In most deep RL-based drug design contributions, the algorithm used is the policy gradient algorithm REINFORCE [31], which uses the gradient ascent to maximize a predefined reward. The update rule of the REINFORCE algorithm consists of maximizing the expected return described in Eq. 4 by iteratively computing its gradient with respect to the model’s parameters. By doing so, the REINFORCE algorithm enforces the generative model to increase the probability of selecting the expectedly high rewarding actions and decrease low rewarding ones.

4 $$J(\theta )=\mathrm{E}[\mathrm{r}(\mathrm{s},\mathrm{a})] =\sum_{t=0}^{T}p({s}_{t},{a}_{t})({r}_{t})\to \mathrm{max }| {\mathrm{s}}_{0}, \theta$$

where θ is the generative model’s set of parameters, E is the mathematical expectation, r is the reward, s is the state (the input character), and a is the action (the output character).

The RL environment in the case of drug design is a program (ML model or simple function) that encapsulates the desired properties. It takes in the molecule given by the generator and outputs a score indicating how much close or far it is from the desired. After multiple trials and errors to set the optimal hyperparameters (learning rate, number of epochs, seeds, etc.), we launched the REINFORCE training loop to bias G towards the desired properties described in “The biassing objectives” section and recorded the results.

Note: (please see Appendix B for the pseudocode of biassing G using RL).

### Biassing G using CRLV

Our proposed approach is simple. It utilizes the same training loop used for the general model G, except that the value of the cross-entropy loss is reduced according to an intermediate test that aims to assess the frequency with which G designs molecules within the desired chemical space. The intuition is that by doing so, negligible loss values are backpropagated through the model, causing it to keep the same set of weights. On the other hand, if the generated molecules are not within the desired space, the loss backpropagated is just the one used in the training of G, which helps in both (i) not forgetting how valid molecules look like as well as (ii) exploring other chemical spaces since the training data is randomly sampled. Finally, we launched the CRLV loop to bias G towards the desired properties described in “The biassing objectives” section and recorded the results.

Note: (please see Appendix B for pseudocode of biassing G using CRLV).

### The biassing objectives

To assess the performance of our approach, we created four benchmarking tasks. We used RL and CRLV biassing techniques at each task to focus G on the desired chemical space. The four benchmarking tasks/objectives are the following:

1. A single objective: logP ≤ 3, one component of the rule of three [9] for orally bioavailable compounds.
2. Multiple structural objectives as described in [13] for molecules that are most likely to be drugs. These are: (i) 2 and 1 for the numbers of aromatic and non-aromatic rings, respectively. (ii) The presence of at least one of the following functional groups: -OH, -COOR, -COOH, or -NH2. (iii) The R-value should be within the intervals of [0.05–0.50].
3. Multiple chemical objectives: the full Lipinski’s rule of three, that is: logP ≤ 3, molecular weight ≤ 480 g/mol, ≤ 3 hydrogen bond acceptors and donors respectively, and ≤ 3 rotational bonds.
4. Objectives (2) and (3) simultaneously.

The last three multi-criteria objectives were ordered according to their frequency in the Chembl21 dataset. We hypothesize that the difficulty of each objective is proportional to its frequency in the training data because less frequent ones will be less sampled and, thus, less likely to be generated by G. The number of molecules that satisfy objectives 2, 3, and 4 in Chembl21 is 365 k, 72 k, and 16 k respectively.

### Metrics

For each of the three models (G, RL-biased-G, and CRLV-biased-G), four metrics were computed:

1. Validity: the fraction (percentage) of chemically valid SMILES among all generated ones, computed using the open-source chemistry library RDKit [25].
2. Novelty: the fraction of novel molecules (i.e., not present in the training dataset) among the valid molecules.
3. Uniqueness: the fraction of molecules after eliminating duplicates among the previously computed novel ones.
4. Internal diversity (intDiv): computed using the open-source library MOSES [22]. It evaluates the chemical diversity of the generated valid, novel, and unique molecules using the Tanimoto similarity index [2]. This metric is bounded between 0 and 1, where closer values to 1 indicate high diversity.

Additionally, a fifth metric was calculated for the biased models only:

(5) Desirability: the fraction of valid, novel, and unique molecules with the desired properties.

## Results and discussion

Note: the benchmarks presented in Tables 1, 2, 3, 4, and 5 are conducted on 10 k molecules generated using the corresponding models. The validity, novelty, and uniqueness are reported in percentages, while desirability is presented in percentages and the actual integer value (Tables 2, 3, 4, and 5). As explained earlier, the internal diversity (intDiv) is a value between 0 and 1, where closer values to 1 indicate high diversity.

**Table 1:** Benchmark results of the general model G

Temperature	Validity	Novelty	Uniqueness	intDiv
1	34.2 ± 5.1	96.5 ± 0.9	99.8 ± 0.7	0.84 ± 0.05
0.80	52.4 ± 4.5	94.2 ± 1.2	98.0 ± 1.8	0.83 ± 0.18
0.60	65.8 ± 3.6	93.3 ± 1.9	97.4 ± 3.2	0.82 ± 0.90
0.50	80.0 ± 2.9	92.5 ± 2.5	88.2 ± 3.9	0.81 ± 1.92
0.40	85.7 ± 1.8	91.8 ± 3.7	65.7 ± 5.0	0.79 ± 2.02
0.20	99.7 ± 0.2	91.0 ± 5.2	11.1 ± 6.8	0.73 ± 3.26

**Table 2:** Benchmark results of the RL-biased-G: last epoch

Objective	Validity	Novelty	Uniqueness	intDiv	Desirability
1	41.09	45.94	39.93	0.79	74.40 = 561
2	99.75	61.83	1.07	0.67	24.24 = 16
3	52.97	25.82	24.70	0.79	59.46 = 201
4	99.96	96.69	0.17	0.65	84.23 = 13

**Table 3:** Benchmark results of the CRLV-biased-G: last epoch

Objective	Validity	Novelty	Uniqueness	intDiv	Desirability
1	70.53	78.16	70.45	0.77	57.00 = 2214
2	54.29	90.38	93.49	0.80	25.54 = 1172
3	61.55	87.08	67.51	0.81	12.01 = 434
4	66.37	86.98	73.94	0.82	1.71 = 73

**Table 4:** Benchmark results of the RL-biased-G: best epoch

Objective	Validity	Novelty	Uniqueness	intDiv	Desirability
1	80.96	80.17	77.44	0.81	41.32 = 2076
2	99.52	91.53	10.60	0.66	66.66 = 644
3	86.73	55.22	51.44	0.80	27.75 = 684
4	98.16	97.65	0.33	0.64	51.04 = 16

**Table 5:** Benchmark results of the CRLV-biased-G: best epoch

Objective	Validity	Novelty	Uniqueness	intDiv	Desirability
1	66.33	86.56	81.60	0.82	68.11 = 3192
2	32.42	97.00	96.43	0.81	42.96 = 1302
3	62.45	88.69	77.48	0.82	22.46 = 964
4	67.21	84.88	74.40	0.80	2.52 = 107

### The general model

Before proceeding forward, we had to assess the effect of the temperature parameter T on the performance of G in order to set an optimal value. As shown in Table 1, the higher T is, the lower the validity. This is because, as stated earlier, high-temperature values tend to equalize the probability distribution of the model's output, yielding an equal likelihood of selecting all characters, hence the high diversity and uniqueness. Our goal in this experiment is to choose a temperature value that compromises all four metrics. Thus, we set T = 0.50. For this chosen T, 80% of the 10 k generated molecules are valid, 94% among the valid ones are novel, and 88% among the valid and novel ones are unique, with a relatively good diversity of 0.81.

### Catastrophic forgetting: RL versus CRLV

Catastrophic forgetting (CF) is the tendency of a machine learning algorithm (e.g., neural network) to forget/change the optimal weights learned for an earlier task (e.g., making valid, novel, and diverse molecules) when trying to learn a new task (e.g., making valid and diverse molecules with desirable properties) sequentially.

Our primary motivation behind developing the new CRLV biassing algorithm is that RL suffers from CF significantly. This is manifested by the low novelty, uniqueness, and diversity when using the last version of the trained RL-biased-G, as presented in Table 2. The RL-biased-G tends to overfit the newly learned policy by frequently generating the same successful molecules, especially for objectives 2 and 4.

On the other hand, because it exploits the same cross-entropy loss used to train G, the CRLV-biased-G was able to retain the learned policy corresponding to generating valid, novel, and diverse molecules when biased for the desired chemical space as listed in Table 3. The intuition of CRLV is that, if the generated molecules are desirable, the loss backpropagated will be sufficiently small to make no (or negligible) changes to the model's parameters, whereas if the generated molecules were not within the desired space, the loss backpropagated will be just the one used for training G earlier. Thus, by alternating between the first task (i.e., to generate valid, novel, and diverse molecules) and the second task (i.e., to generate valid, novel, diverse, and desirable molecules), the CRLV technique enforces the biased model to learn an optimal compromise, and therefore, increases desirability while still avoiding the catastrophic forgetting. Moreover, the run-to-run variance in the desirability was higher for RL, specifically, ± 19.87%, ± 11.16, ± 10.51%, and ± 14.92% for objectives 1, 2, 3, and 4, respectively, versus ± 6.73%, ± 4.44%, ± 3.81%, and ± 0.75% for CRLV.

### Further improving the performance

In Tables 2 and 3, the results concern the last version of the biased models, i.e., the one given by the last epoch of the biassing loop. In Tables 4 and 5 on the other hand, we propose to record the best version of the models during the biassing loop, i.e., the one yielding the highest number of desirable molecules. This is accomplished according to the following intermediate test: at each epoch, we use the current version of the model to generate 'k' molecule (k = 20 in our experiment) and only save this version of the model if it surpasses the previous one in the desirability.

Tables 4 and 5 summarize the results of biassing G using RL and CRLV, which show significant improvement. Among the 10 k generated molecules, the RL-biased-G yielded 2076, 644, 684, and 16 valid, novel, and unique molecules that satisfy objectives 1, 2, 3, and 4, respectively. While the CRLV-biased-G managed to generate more desired molecules, namely, 3192, 1302, 964, and 107 valid, novel, and unique molecules that satisfy objectives 1, 2, 3, and 4, respectively. This proves that the performance can be improved in both approaches if the recorded model during biassing is the one yielding the highest number of desirable molecules as opposed to the one given by the last epoch. Further, it reaffirms that supplemental epochs in the fine-tuning phase may result in significant overfitting, thus, stopping when no improvement is made is highly recommended. Finally, the run-to-run variance associated was reduced to ± 5.22%, ± 4.56, ± 2.13%, and ± 0.82% for objectives 1, 2, 3, and 4, respectively for RL, versus ± 4.64%, ± 2.87%, ± 1.93%, and ± 0.71% for CRLV.

As a final note regarding the results of Tables 1, 2, 3, and 4, we reported the desirability in terms of percentages as well as the actual integer values. We did this on purpose since percentages might be misleading. For instance: if out of the 10 k generated molecules, only 5 made it to the uniqueness phase, and these 5 molecules are all desirable, then the percentage of desirability will be 100%, which might mislead the reader into thinking that this approach is the optimal one. Indeed, this work demonstrates exactly this issue for the RL-biased-G. On the other hand, our proposed approach (i.e., CRLV) can generate diverse sets of valid molecules, therefore, many of them make it to the uniqueness phase, and thus, even with seemingly low desirability percentages compared to RL-biased-G, it will be higher in terms of the actual numbers. For example: if out of the 10 k generated molecules by the CRLV-biased-G, 150 made it to the uniqueness phase, even with only 10% desirability, the actual number (i.e., 15) will still be higher than that of the RL-biased-G example of 100% in percentage and 5 for the actual number. Similarly, in the results regarding objective 4 in Tables 4 and 5 for instance, although the percentage of desirability for RL is 51.04%, it is only taken from 31 unique molecules, thus, yielding 16 desirable ones. On the other hand, for CRLV, the percentage of desirability was lower (2.52%). However, this percentage is given out of 4244 unique molecules, resulting in 107 desirable ones. This specific issue is the main problem addressed in the present paper, that is, the goal of the CRLV algorithm is to generate more desirable molecules with **optimal metrics tradeoff**, that is, instead of maximizing validity and desirability percentages while sacrificing other metrics (uniqueness and diversity) as the case for RL, find a compromise between all metrics to ultimately have more desirable molecules as in CRLV.

### Hyperparameters tuning

Besides the CF problem, RL could not generate molecules that satisfy objective 4 with a 0% desirability using the default hyperparameters as opposed to CRLV. Therefore, a range of hyperparameter sets was tested, and the best set was chosen for the model, whose results are presented in Tables 2 and 4. In Appendix D, we present the conducted study to define the optimal hyperparameters for the RL biassing loop.

### RL can focus, but it does not imply maximization

**Fig. 2:**
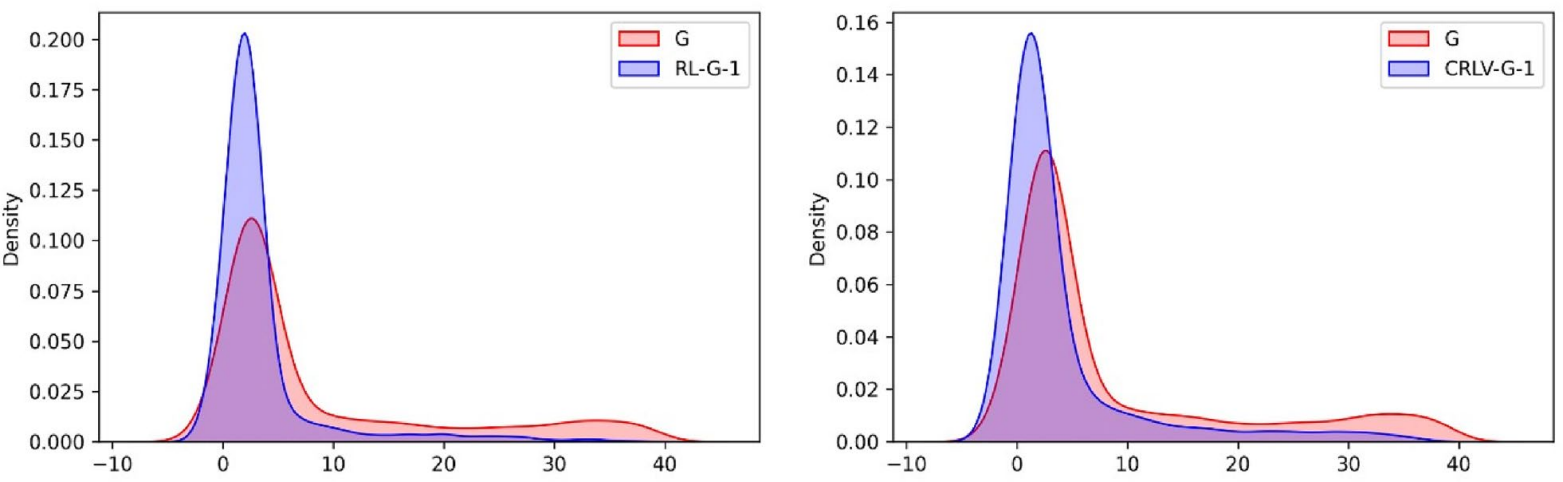
logP distribution for molecules generated by G versus RL-biased-G and CRLV-biased-G respectively

We benchmarked the general model G against the four objectives (Appendix F: Table 6) to assess the degree to which the desirability has increased after the biassing. For instance, G can generate about **2300** molecules satisfying objective 1, that is, more than that of the best RL-biased-G (**2076**). This led us to inspect whether RL has demonstrated any property shift at all. In Fig. 2, we illustrate the kernel density estimation (KDE) plots to demonstrate the shift of the property optimized, in this case, objective 1 (i.e., logP ≤ 3). Although RL-biased-G has successfully shifted a high percentage of its generated molecules toward the desired objective, it was not successful at maximizing that percentage with respect to what the general model (G) is already capable of. This is due to the relatively low novelty, uniqueness, and diversity associated with RL, which eliminates many generated molecules. On the other hand, CRLV-biased-G can generate about **3192** desirable molecules, meaning that it was able to both shift the distribution as well as maximize its quantity. CRLV ensures either comparable or better performance to that of G because of its conditional loss function that encompasses both the task of G and the desired property optimization task. In summary, RL can focus the policy of a general model such as G, by making it always generate molecules with the desired properties, however, it is indifferent to whether these molecules are diverse or not, which makes it good for biassing, but not necessarily for maximization. This specific investigation is what was missing from many related contributions, that is, only the property distribution shift that was presented, without assessing the capability of the biased model to maximize the generated desired molecules. In Appendix F, we further present the distribution shift for objective 2 (Fig. 11) and objective 3 (Fig. 12) along with the benchmarking results of G.

### The possibility of using both methods jointly

**Fig. 3:**
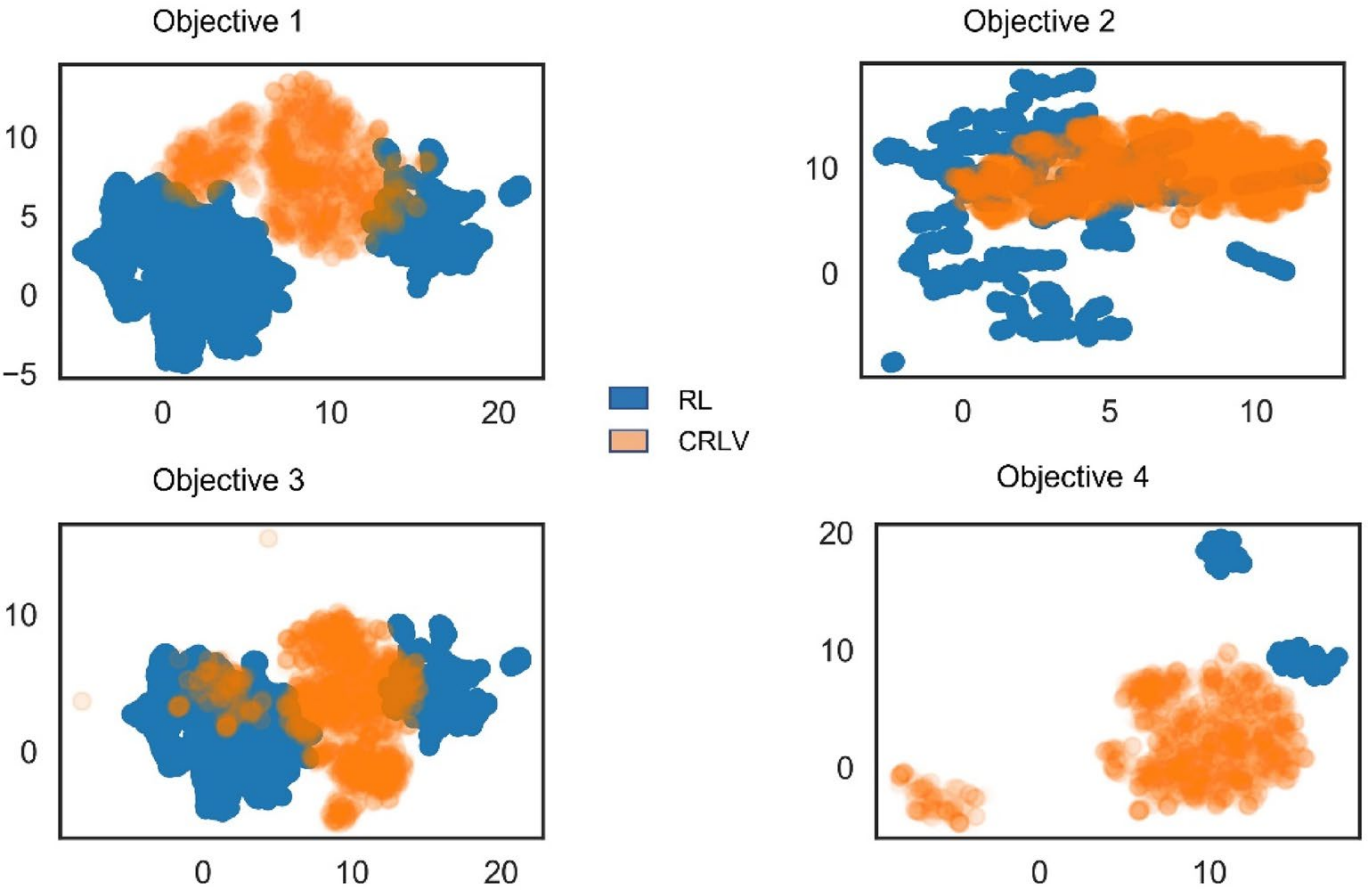
UMAP plots of RL-biased-G molecules and CRLV-biased-G molecules for all four objectives

This subsection aims to determine whether RL-biased-G and CRLV-biased-G can be used jointly. We evaluated the number of intersections for all four objectives by generating 10 k molecules using the corresponding model of each biassing technique, removing duplicates, and computing the number of similar molecules between both sets. The percentages of intersection were 3.62%, 0.29%, 5.06%, 0.01% for objectives 1, 2, 3, and 4 respectively. The low intersections suggest that the two techniques can be used jointly to increase desirability while retaining diversity. Moreover, it led us to question to which degree the learned chemical spaces by the two models differ. For this, we decided to explore the uniform manifold approximation and projection (UMAP) plots of Morgan circular fingerprints [15] of length 1,024 bits and radius of 2 bonds of molecules generated by each model as presented in Fig. 3. Conformally, the chemical spaces learned by the two models for each objective do not significantly overlap. This demonstrates that RL and CRLV can be used not only to make novel, chemically valid, desirable drugs but also to augment drug libraries that contain a small number of samples by using them jointly.

### The difficulty of the defined objectives

In Table 2, CF was more notable for objectives 2 and 4 than objectives 1 and 3, suggesting that structural objectives may be more challenging to learn than chemical objectives. This was also remarked in Table 4 (for RL), as uniqueness was much lower for objectives 2 and 4, while in Table 5, objective 2 (for CRLV), the validity dropped considerably, compared to other objectives. In this subsection, we explore the main features that may contribute to the difficulty of an objective in this context.

**Fig. 4:**
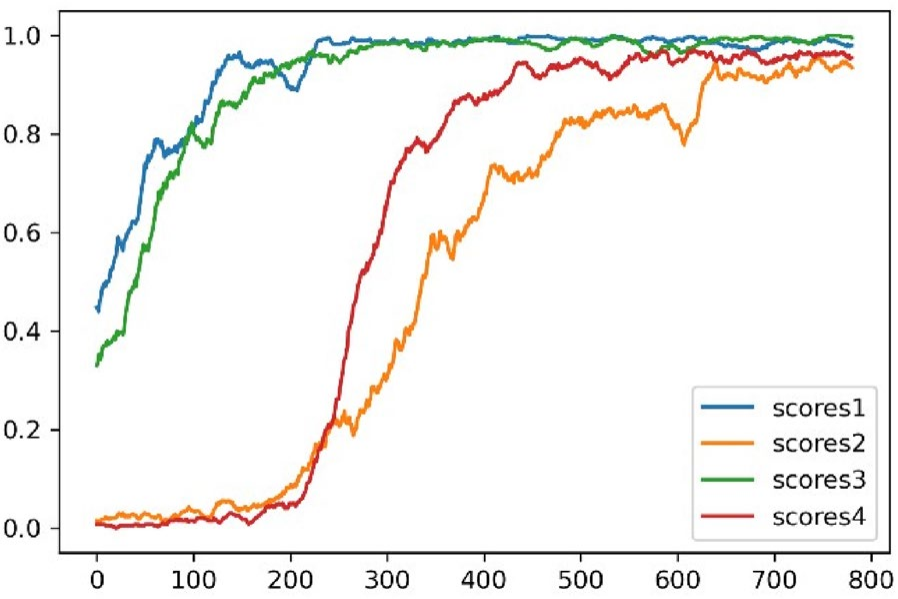
The learning curves (scores) of the RL-biased-G for all four objectives

We stated earlier that the difficulty of target molecules is proportional to their frequency in the training dataset. However, the learning curves of the RL-biased-G of the four objectives illustrated in Fig. 4 show a somewhat surprising finding: the model needed more time to learn objective 2 than objective 4, although the latter is less present in the training dataset. We hypothesized that the cause of this finding might be due to the structural variance (SV) related to objective 2. We consider SV a parameter that should be proportional to the number of unique characters required to make a target molecule. The intuition behind our hypothesis is that the more a target molecule requires many diverse characters, the higher the likelihood of making validity-related mistakes while trying to generate such molecules.

The following are examples of molecules with low SV and high SV, respectively from the Chembl21 dataset:

Low SV molecules:

- **M-low-1**: Nc1cccc2ncccc12
- **M-low-2**: COc1ccccc1CCN

High SV molecules:

- **M-high-1**: [K +].[O-]S(= O)(= O)c1ccccc1C(= O)n2cc(C(= O)c3ccn4[C@H](SCc34)c5cccnc5)c6ccc(cc26)c7ccc(F)cc7
- **M-high-2**: [Na +].CO\\N = C(/C(= O)N[C@H]1[C@H]2SCC(= C(N2C1 = O)C(= O)[O-])COC(= O)C)\\c3csc(N)n3

For this purpose, we introduced a new metric to quantify SV for each of the multi-objectives 2, 3, and 4 as described in Eq. 5, which is equal to the number of unique characters (i.e., after eliminating duplicates) in the SMILES representing the molecule divided by 100, where 100 is the global maximum length of all molecules used in our study. Dividing by the global maximum length; instead of each molecule's length; sets a common reference between molecules and thus, allows for relative comparisons. For instance, the SV of **M-low-1** and **M-high-1** given above, if we divide by the molecule's length, are 0.33 and 0.23, respectively. This is incorrect and does not reflect our definition of SV. Nevertheless, when dividing by the global maximum length, **M-low-1** and **M-high-1**'s SV is 0.05 and 0.24, respectively.

SV is higher when many unique characters constitute the SMILES and lower otherwise**.**

5 $$SV(SMILES)=\frac{number\, of\, unique\, characters}{max\, length}$$

**Fig. 5:**
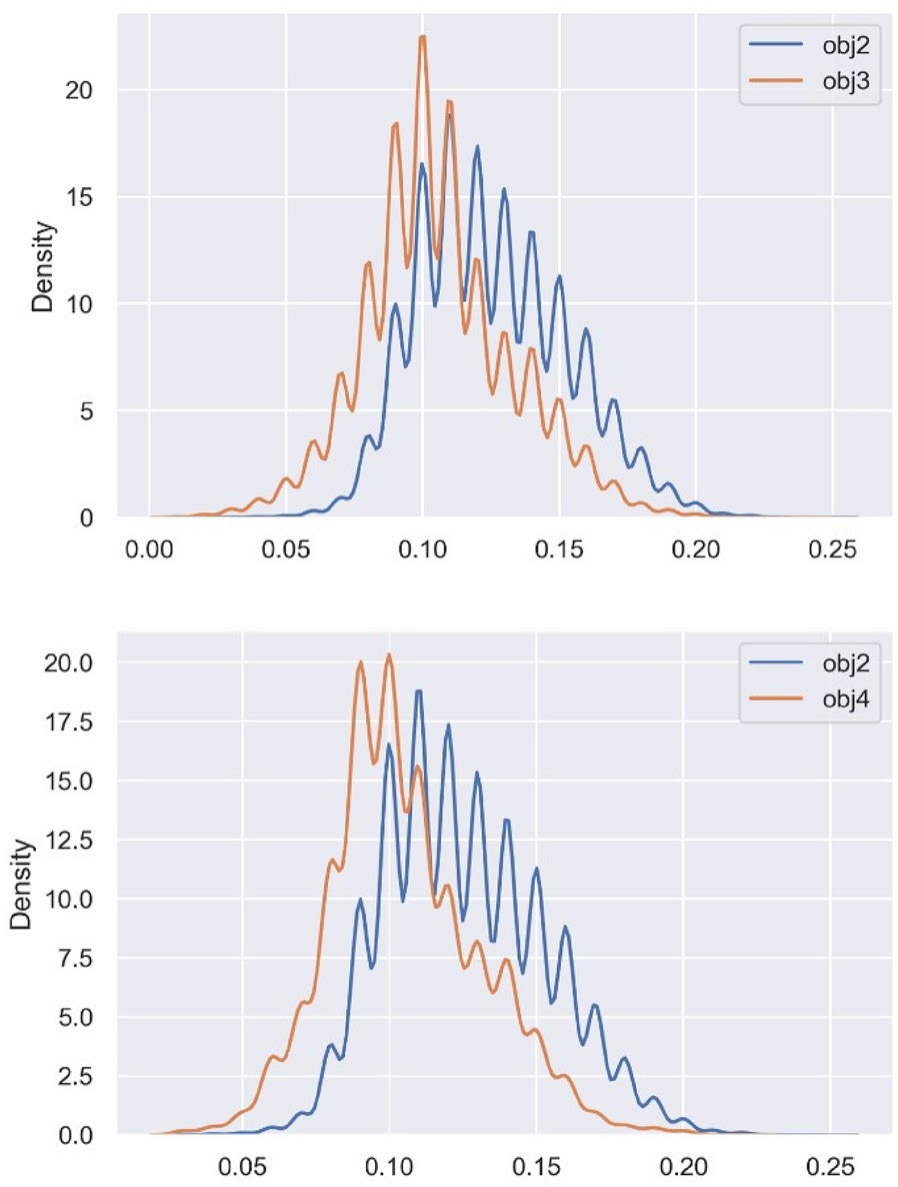
The structural variance of molecules satisfying objective 2 versus molecules satisfying objectives 3 and 4, respectively

**Fig. 7:**
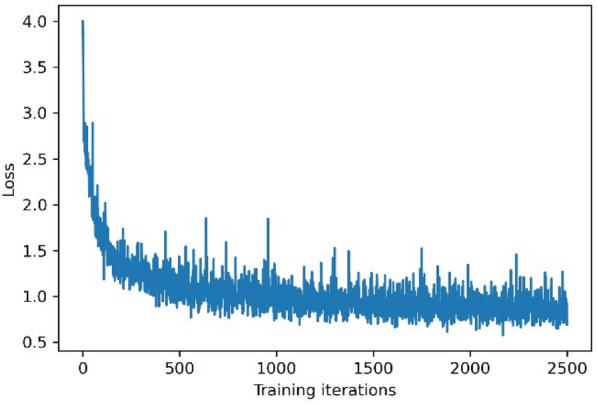
The learning curve of the general model G

**Fig. 9:**
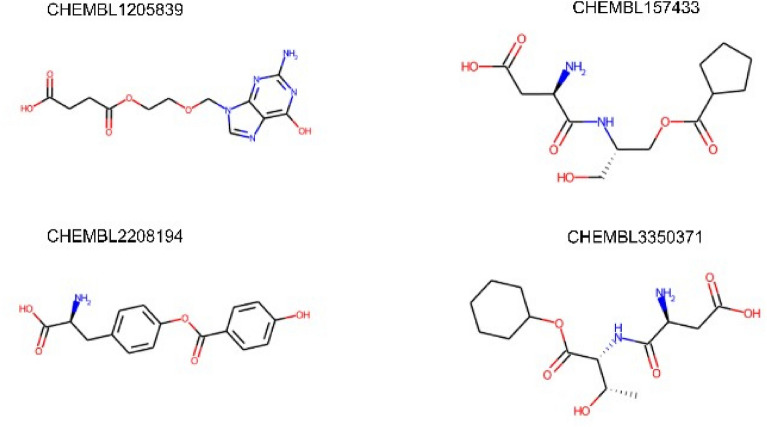
Four examples of molecules that satisfy objective 5 from Chembl21

**Fig. 10:**
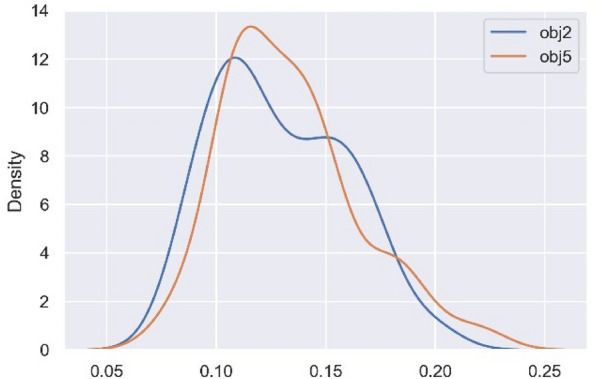
Objective 5 presents more SV than objective 2

**Fig. 11:**
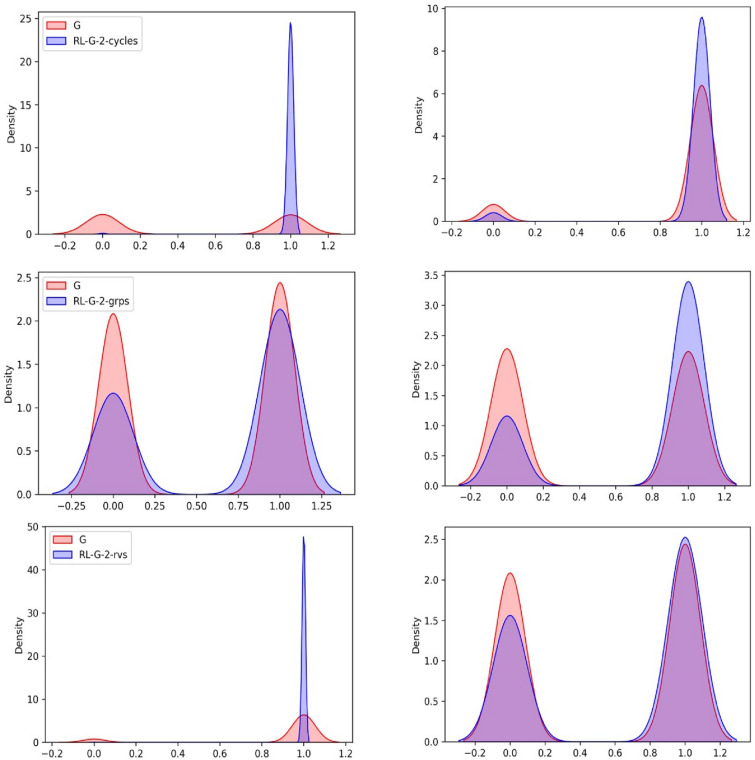
Property shift demonstration for objective 2. We categorically plotted the distribution of molecules, that is, molecules with optimized properties (number of rings (cycles), number of functional groups, and the R-value), are positioned around x = 1, whereas non-optimized molecules are placed on x = 0. Here we can see another reason why RL-biased-G did not generate many successful molecules for objective 2, which is the inability to design molecules with the required functional groups (second sub-figure on the left), as opposed to the number of the rings and the R-value that were highly optimized only

**Fig. 12:**
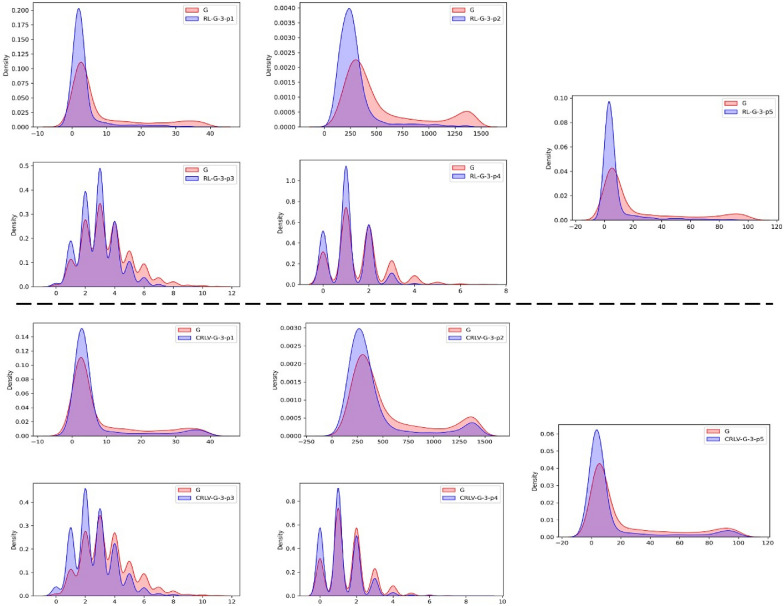
Property shift demonstration for objective 3

We computed the SVs for molecules from the Chembl21 dataset that satisfy objectives 2, 3, and 4 and plotted them as KDE distributions in Fig. 5. The result confirms our hypothesis: objective 2 requires higher SV than objectives 3 and 4. Further, we specified the fifth objective and evaluated the ability of both techniques to bias G toward it. Objective 5 is defined the same as objective 2, except that “all functional groups are required to be present” instead of “at least one of them”. That is: (i) 2 and 1 for the numbers of aromatic and non-aromatic rings respectively, (ii) the presence of all following functional groups: -OH, -COOR, -COOH, or -NH2 in the same molecule. (iii) The R-value should be within the intervals of [0.05–0.50]. Examples of such molecules from Chembl21 are depicted in Appendix E: Fig. 9. The number of molecules in the Chembl21 dataset that satisfy objective 5 is only 76 out of the 1.5 M molecule, and the structural variance (illustrated in Appendix E: Fig. 10) related to this objective is higher than that of objective 2. Biassing G towards a chemical space containing molecules satisfying objective 5 has failed for RL and CRLV with 0% desirability. This issue is of great importance and should be addressed in future work.

## Conclusion

In summary, the purpose of the present paper is to support four main findings: (i) Simple CRLV may be better than RL (with the REINFORCE algorithm) for biassing a general molecular generator G. This is because it allows not only to focus G’s policy on the desired chemical space but also to maximize the number of desired molecules with sufficient diversity. (ii) Further, because it utilizes a similar loss used for training G, catastrophic forgetting is negligible in CRLV compared to RL. (iii) Nevertheless, CRLV and RL can be used jointly to increase the desirability given the low percentage of intersections between the two. (iv) Finally, as a side note, yet an important one, the difficulty of an objective is proportional to, not only its frequency in the training dataset but also the associated structural variance SV.

Although most current related contributions use RL with the REINFORCE algorithm for biassing molecular generators towards the desired chemical space, the present study shows that it may not be an optimal approach. Results in Table 2, 3, 4, and 5 evidence that the simple CRLV method outperformed it. Many issues that might cause the relatively suboptimal performance of RL-biased-G are already addressed in core Deep RL papers. Indeed, it is well known that REINFORCE is very sensitive to hyperparameter tuning due to the noisy estimate of the gradient and the unclear credit assignment. That is, rewards are only given at the end of each episode, while each action (i.e., output character) will affect the overall result differently. The RL community proposed many solutions for these problems, such as the proximal policy optimization (PPO) algorithm [28]. The critical contribution of PPO is to ensure that a new policy update does not change it too much from the previous policy. This leads to less variance and smoother training and guarantees that the agent does not go down an irremediable path while efficiently exploring the space. Such new sophisticated algorithms will be the object of future investigation.

We believe this study is directly helpful for many cheminformatics applications, namely, de novo drug design, as it provides a novel technique for generating multi-objective optimized drugs. Besides, RL and CRLV can also be used for data augmentation to increase a set of molecules for a machine learning-based bioinformatics task such as drug-target interaction (DTI) and drug target affinity (DTA) prediction where data is often imbalanced [19].

Finally, we hope these results will motivate other future improvements toward a more efficient exploration of the vast chemical space, especially when many constraints must be satisfied and representative training data is lacking, as in the case of objective 5 discussed. In this respect, the proposed CRLV biassing technique can be considered an additional baseline for future benchmarks.

## Figures and Tables

**Fig. 6:**
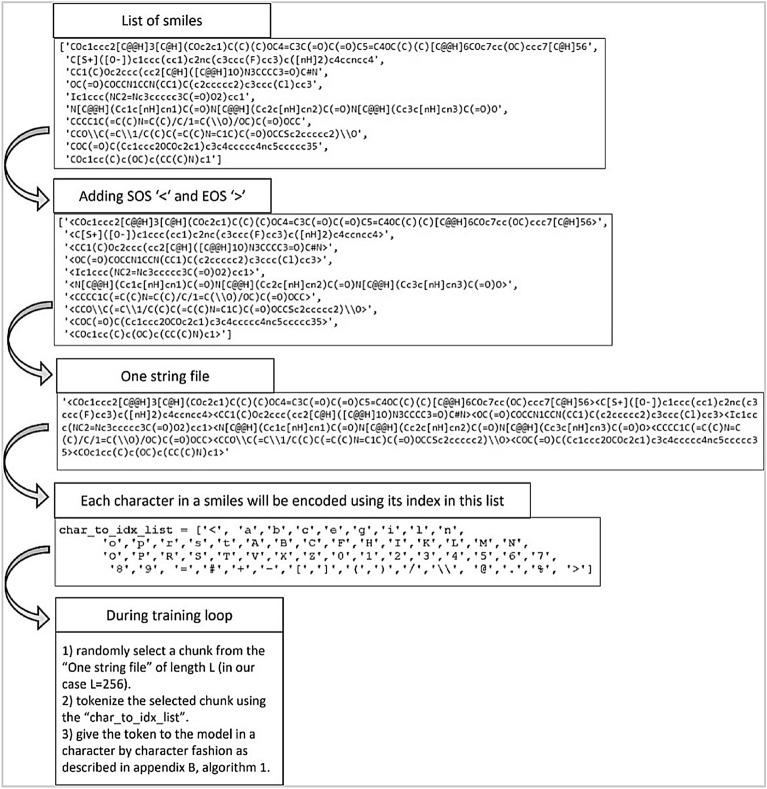
The steps followed for data preparation

**Fig. 8:**
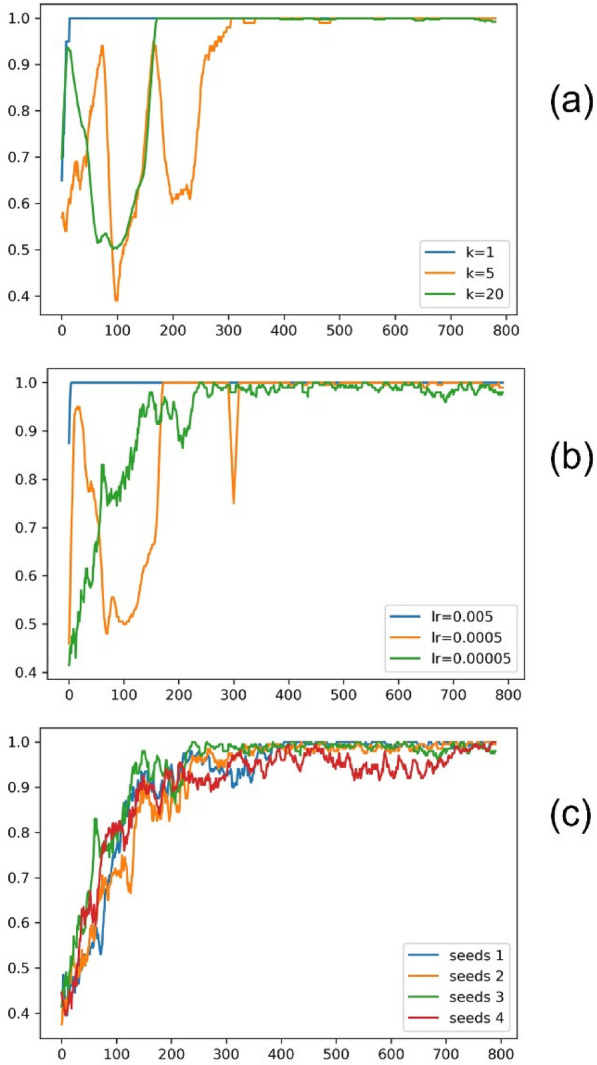
Biassing G using RL for objective 1: **a** trying different k episodes, same learning rate used for G of 0.0005, and default random seeds. **b** trying different learning rates, k = 20, and default random seeds. **c** trying different random seeds, k = 20 and a learning rate of 0.00005

## Data Availability

All code and data used in this work are accessible online via an open-source repository at: https://github.com/amine179/DrugDesign.

## Appendix A: Data preparation

The Chembl21 dataset contains a list of 1.59 M SMILES. Molecules with a size (number of characters in the SMILES notation) above 100 were excluded leaving us with a list of 1.49 M SMILES. We then added to each molecule two symbols for the start (SOS) and end (EOS) of the sentence, represented respectively by ' < ' and ' > '. This helps organize data for the third step, which consists of merging all molecules into one giant string file that will be used to sample training data. After defining the set of all characters present in the dataset at the fourth step, the training loop can be commenced according to algorithm 1 in Appendix B. This encompasses (i) a random selection of a chunk of length L (in our case, L = 256) from the giant string file, (ii) tokenizing the selected chunk by mapping each character in the chunk to its index in the set of characters, and finally, (iii) giving the tokenized chunk as input to the model in a character-by-character fashion. All discussed steps are illustrated in Fig. 6.

## Appendix D: Hyper-parameters tuning for RL-based biassing G

We conducted a comprehensive experiment to uncover the optimal hyperparameters for RL and used them for comparison against CRLV. As reported in Fig. 8, Keeping the same learning rate used to train G yields a high variance for RL. This led us to decrease the learning rate by ten (i.e., from 0.0005 to 0.00005), significantly improving the stability. Moreover, learning from multiple episodes (e.g., k = 20) helped stabilize the run-to-run variation and performance of the RL-biased-G. Different random seeds did not considerably affect the learning curve after setting the learning rate and the number of learning episodes to 0.00005 and 20, respectively. Lower values of the learning rate or higher values of the number of learning episodes than those fixed did not improve the learning anymore. Therefore, we considered the best hyperparameters for RL-biased-G, whose results are reported in Table 2, to be the ones yielding stable training results and higher desirability, with a permitted minimum diversity of 0.79.

## Appendix F: Distribution shift for multi-objective 2 and 3, and the benchmarking results of G

**Table 6:** Benchmark results of the general model G for all four objectives

objective	Validity	Novelty	Uniqueness	intDiv	Desirability
1	77.53	92.63	79.23	0.80	41.90 = 2385
2	78.62	92.16	81.02	0.80	17.04 = 996
3	81.17	91.73	81.30	0.80	9.81 = 594
4	77.01	92.28	78.44	0.81	1.25 = 69

## Abbreviations

CF: Catastrophic forgetting
CRLV: Conditional reduction of the loss value
G: General molecular generator
GRU: Gated recurrent unit
KDE: Kernel density estimation
LogP: Lipophilicity
RL: Reinforcement learning
SMILES: Simplified molecular line-entry system
SV: Structural variance
UMAP: Uniform manifold approximation and projection
